# Structure of Aurora B–INCENP in complex with barasertib reveals a potential transinhibitory mechanism

**DOI:** 10.1107/S2053230X14002118

**Published:** 2014-02-19

**Authors:** Fabio Sessa, Fabrizio Villa

**Affiliations:** a Department of Experimental Oncology, European Institute of Oncology, Via Adamello 16, 20139 Milan, Italy

**Keywords:** Aurora kinases, barasertib, cancer, PKA, small-molecule inhibitors

## Abstract

The Aurora family is a well conserved and well characterized group of serine-threonine kinases involved in the normal progression of mitosis. The deregulation of Aurora kinases impairs spindle assembly, checkpoint function and cell division. To date, many small molecules that compete with ATP for binding to Aurora kinases have been developed and characterized. Here, the first structure of the *Xenopus laevis* Aurora B–INCENP complex bound to the clinically relevant small molecule barasertib was determined. The binding properties of this inhibitor to the Aurora B active site are analyzed and reported. An unexpected crystal-packing contact in the Aurora B–INCENP structure coordinated by an ATP analogue is also reported, in which the INCENP C-­terminus occupies the substrate-binding region, resembling the protein kinase A inhibitory mechanism.

## Abstract 2

Two structures of Aurora B kinase bound to the specific inhibitor barasertib and to a nonhydrolyzable nucleotide provide evidences for a potential transinhibitory mechanism.

## 1. Introduction   

The Aurora proteins are a small family of serine/threonine kinases that are expressed during mitosis and have been suggested to be attractive drug targets (Carpinelli & Moll, 2008 ▶; Andrews, 2005 ▶). Mammalian cells possess three Aurora kinase isoforms (Aurora A, B and C) that exhibit different subcellular localizations and have distinct functional roles (Carmena & Earnshaw, 2003 ▶). Together with INCENP, borealin and survivin, Aurora B kinase forms the chromosome passenger complex (CPC; Carmena *et al.*, 2012 ▶). The most conserved C-terminal region of INCENP (the so-called IN-box) is sufficient to bind and stimulate the catalytic activity of Aurora B (Honda *et al.*, 2003 ▶; Sessa *et al.*, 2005 ▶). The CPC complex is involved in numerous mitotic functions, and the timing and mechanisms of each of these events are carefully regulated by its localization within mitosis (Carmena *et al.*, 2012 ▶). The selection of Aurora substrates is also aided by a precise and exclusively unique phosphorylation motif (Alexander *et al.*, 2011 ▶).

**Figure 1:**
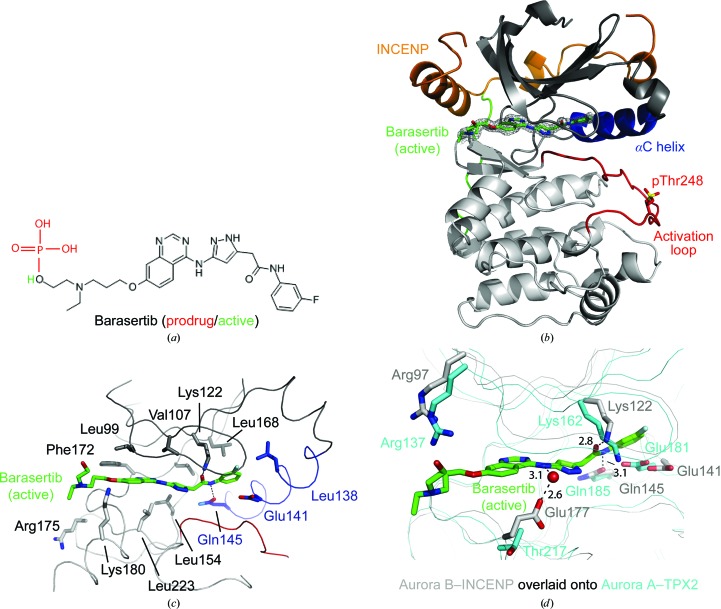
Binding of barasertib to the ATP-binding pocket of Aurora *B*. (*a*) Chemical structure of barasertib prodrug (red) and the active form (green). (*b*) The three-dimensional structure of Aurora B in cartoon representation, illustrating the N-terminal small lobe (grey), the C-terminal helical large lobe (white), the short C-terminal extension (green), the αC helix (blue) and the activation loop (red). INCENP (orange) crowns the small lobe of Aurora B, stabilizing an active conformation of the kinase. The active form of barasertib (sticks) and its unbiased *F*_o_ − *F*_c_ OMIT electron-density map (grey mesh) occupy the ATP-binding pocket at the interface between the small and large lobes. (*c*) Stick representation of the interaction between the active form of barasertib (green) and selected residues of Aurora B. (*d*) Structure superimposition of the Aurora B–INCENP–barasertib active form complex (grey) with the Aurora A–TPX-2 complex (cyan; PDB entry 1ol5; Bayliss *et al.*, 2003 ▶). O, N and S atoms are shown in red, blue and yellow, respectively. Hydrogen bonds are shown as dashed lines and bond lengths are indicated in Å.

As expected, deregulation of Aurora kinases has drastic intra­cellular consequences (Meraldi *et al.*, 2004 ▶), rendering these kinases attractive drug targets for small-molecule inhibitor development, targeting the active-site binding pocket which binds the ATP substrate (Andrews, 2005 ▶). Several small-molecule inhibitors of Aurora kinases have been developed for use as anticancer agents, some of which have progressed to early clinical evaluation (Carpinelli & Moll, 2008 ▶). Barasertib is a highly potent and selective inhibitor of Aurora B (*K*_i_ = 1 n*M*) in comparison with Aurora A (*K*_i_ = 1.4 µ*M*) and has high specificity *versus* a panel of 50 other kinases (Mortlock *et al.*, 2007 ▶). Barasertib is a prodrug of a pyrazoloquinazoline Aurora kinase inhibitor (Fig. 1 ▶ *a*) and is rapidly converted to the active drug in plasma (Mortlock *et al.*, 2007 ▶; Fig. 1 ▶ *a*). Consistent with the inhibition of Aurora B kinase, the addition of barasertib to tumour cells *in vitro* induces chromosome misalignment, prevents cell division and consequently reduces cell viability and induces apoptosis (Yang *et al.*, 2007 ▶).

Here, we report the crystal structure of the *Xenopus laevis* Aurora B–INCENP complex bound to the active form of barasertib. We also report the structure of the same complex bound to a nonhydrolyzable nucleotide that induces the formation of a crystal contact mediated by the substrate-recognition region of Aurora B and a neighbouring INCENP molecule.

## 2. Materials and methods   

### 2.1. Protein expression, crystallization and structure determination   

#### 2.1.1. Aurora B–INCENP–barasertib complex   

The conditions for the expression, purification and structure determination of the *X. laevis* Aurora B–INCENP complex have been described previously (Sessa *et al.*, 2005 ▶). Crystals obtained by microseeding were gradually transferred into cryo-buffer (19% PEG 400, 100 m*M* bis-tris propane pH 6.5, 2 m*M* TCEP) and then incubated with 1/100 (*v*/*v*) of a 10 m*M* solution of barasertib (AZD1152-HQPA, Selleck Chemicals) dissolved in DMSO. After 16 h incubation with the inhibitor, the crystals were flash-cooled. X-ray diffraction data were collected from a single crystal on beamline PXIII at the Swiss Light Synchrotron (SLS), Villigen, Switzerland. Data processing was carried out with *XDS* (Kabsch, 2010 ▶) using the *xia*2 automated data-reduction platform (Winter *et al.*, 2013 ▶). To refine the orientation and location of the molecules, rigid-body refinement with *REFMAC* (Murshudov *et al.*, 2011 ▶) was employed using the Aurora B–INCENP–hesperadin complex (PDB entry 2bfy; Sessa *et al.*, 2005 ▶) as a model. Iterative model building was carried out with *Coot* (Emsley *et al.*, 2010 ▶) and *REFMAC* (Murshudov *et al.*, 2011 ▶), resulting in a good final model (Table 1 ▶).

#### 2.1.2. Aurora B–INCENP–AMP-PNP complex   

Co-crystals of Aurora B–INCENP in complex with AMP-PNP (Roche) were obtained by incubating the protein in the presence of an excess of the nucleotide analogue (1 m*M* final concentration) for 1 h at 4°C prior to crystallization. Crystals grew in a similar condition and crystal handling was carried out as described above. X-ray diffraction data were collected from a single crystal on beamline PXIII at the SLS. Data processing and refinement were carried out as described for Aurora B–INCENP–barasertib, resulting in a good final model (Table 1 ▶).

## 3. Results and discussion   

### 3.1. Crystal structure of Aurora B bound to the highly specific inhibitor barasertib   

Despite the high reported specificity of barasertib towards Aurora B (Mortlock *et al.*, 2007 ▶), structural data describing the interaction between barasertib and Aurora B are still lacking. In this context, we sought to investigate this inhibition mechanism at the atomic level. For this purpose, we employed the previously described *X. laevis* Aurora B–INCENP complex as a structural template (Sessa *et al.*, 2005 ▶). The sequence identity between the catalytic kinase domains of *X. laevis* and *Homo sapiens* Aurora B is significantly high. We determined the crystal structure of the *X. laevis* Aurora B–INCENP complex at 1.5 Å resolution, highlighting the binding of the active form of barasertib to the ATP active site (Fig. 1 ▶). Barasertib occupies the deep ATP-binding cleft at the interface between the small and the large lobes of the kinase (Fig. 1 ▶ *b*), and its binding does not result in any significant conformational changes relative to the unbound kinase, which crystallizes in a partially active state (Sessa *et al.*, 2005 ▶). Barasertib is bound at the ATP-binding site of the Aurora B kinase domain, where it spans the entire length of the cavity from the hinge-loop to the αC helix (Fig. 1 ▶ *b*). Stacking interactions with the side chains of Leu99, Phe172, Leu223, Leu154 and Val107 hold the quinazoline and anilino moieties of barasertib in place (Fig. 1 ▶ *c*). Furthermore, the carbonyl group and the amino group of the 3-­fluoroaniline are engaged in hydrogen bonding to Lys122 and Gln145, respectively (Fig. 1 ▶ *c*). The 3-fluoroaniline group is housed in a hydrophobic pocket formed by Leu138 and Leu168, disrupting the ion pair formed between the conserved Lys122 and Glu141 residues (Fig. 1 ▶ *c*).

### 3.2. Unravelling barasertib selectivity towards the Aurora B isoform   

Of the many Aurora B inhibitors that have been reported in the literature, in recombinant enzyme assays barasertib shows a higher selectivity, with more than a 1000-fold greater potency, for Aurora B than for Aurora A, with IC_50_ values of 1 n*M* and 1.4 µ*M*, respectively (Mortlock *et al.*, 2007 ▶). In order to elucidate the structural basis that underlies this selectivity, we superimposed the Aurora B–INCENP–barasertib complex with the Aurora A–TPX-2 complex (Bayliss *et al.*, 2003 ▶; Fig. 1 ▶ *d*). Although the ATP-binding cleft is highly conserved between the two enzymes, the biochemical data clearly show strong selectivity of barasertib towards Aurora B (Mortlock *et al.*, 2007 ▶; Fig. 1 ▶ *d*). The *X. laevis* Aurora B residues engaged in hydrogen-bonding to barasertib within the active site are well conserved in the ATP-binding site of *H. sapiens* Aurora A (Fig. 1 ▶ *d*) and are therefore likely to interact with the small molecule in a similar manner. The only observed difference is the hydrogen-bonding of a negatively charged residue (Glu177^Aurora B^) to barasertib through a well defined water molecule (Fig. 1 ▶ *d*). In Aurora A, this residue is replaced by a smaller threonine residue (Thr217^Aurora A^) that is too distant to influence the inhibitor binding (Fig. 1 ▶ *d*). Both Glu177^Aurora B^ hydrogen-bonding and van der Waals contributions might justify the selectivity of barasertib towards Aurora B. Previous work has also established the key role of Thr217^Aurora A^ in determining the selectivity of the small molecule MLN8054 towards Aurora A (Dodson *et al.*, 2010 ▶). Interestingly, a well positioned arginine residue (Arg97^Aurora B^, Arg137^Aurora A^) is ready to stabilize the hydroxyl functional group of the active molecule in plasma in both Aurora isoforms (Fig. 1 ▶ *d*).

### 3.3. An unexpected crystal contact occupies the Aurora B substrate-binding region   

**Figure 2:**
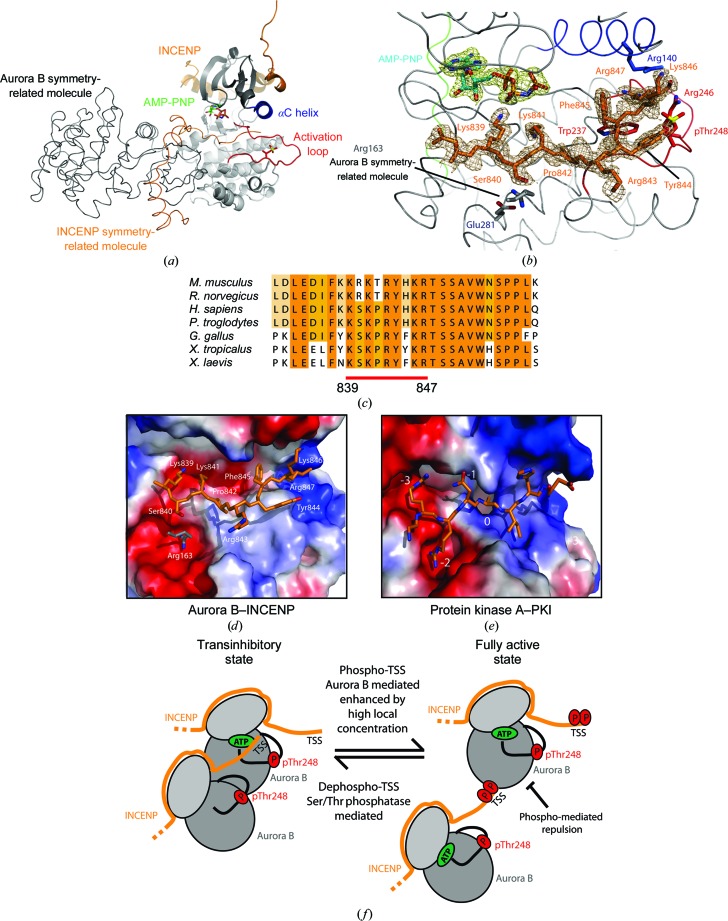
The INCENP C-terminal extension occupies the Aurora B substrate-recognition surface. (*a*) The arrangement of Aurora B–INCENP molecules in the asymmetric unit is shown in cartoon and ribbon format in grey (Aurora B) and orange (INCENP). The C-terminal portion of INCENP packs against the Aurora B large C-lobe. (*b*) Details of the INCENP IN-box (orange) molecular interactions with the Aurora B kinase domain (grey). Calculated electron-density maps for the AMP-PNP and for the INCENP segment are drawn in green and brown, respectively. O, N and S atoms are shown in red, blue and yellow, respectively. (*c*) Multiple sequence alignment of the INCENP C-­terminal region from different species. The alignment is colour-coded based on conservation. A solid red bar indicates the INCENP stretch occupying the Aurora B substrate-recognition surface. Numbering is according to the *X. laevis* sequence. (*d*, *e*) Surface representations of Aurora B with INCENP (*d*) and of protein kinase A with PKI (PDB entry 1atp; Zheng *et al.*, 1993 ▶) (*e*). Surfaces are coloured according to the electrostatic potential distribution of the kinases, ramped from blue (positive) to red (negative). (*f*) Schematic representation of an Aurora B potential transinhibitory model proceeding through an intermolecular mechanism, which may be enhanced by a high local protein concentration. This transinhibition mechanism may be relieved by a pTSS–pThr248 repulsion and lead to full activity of Aurora B. A Ser/Thr phosphatase can then revert the full Aurora B activity to a transinhibited state.

During refinement of the Aurora B–INCENP–barasertib structure, we noticed extra electron density corresponding to a well ordered arginine residue, Arg847^INCENP^, in close proximity to the activation loop (data not shown). We sought to investigate this further, and grew crystals of Aurora B–INCENP in the presence of a nonhydrolyzable ATP analogue (AMP-PNP). The 1.7 Å resolution electron-density map revealed that the nucleotide phosphate groups stabilized an unexpected crystal-packing contact (Fig. 2 ▶ *a*). The C-terminus of a neighbouring INCENP molecule occupies the Aurora B substrate-binding region of an adjacient symmetry mate (Fig. 2 ▶). The INCENP C-terminal peptide was found in an extended conformation engaged with the large Aurora B C-terminal lobe (Fig. 2 ▶ *a*). INCENP residues from Lys839^INCENP^ to Arg847^INCENP^ structurally mimic a substrate peptide bound to the large C-lobe of the catalytic domain of the Aurora B kinase domain (Fig. 2 ▶ *a*). The well defined electron-density map clearly reveals contiguity of the main chain of the INCENP C-­terminus up to the last residue of the crystallized construct (Arg847^INCENP^; Fig. 2 ▶ *b*). The structure of the kinase domain was not perturbed and it adopts a highly similar overall conformation to the previous published structures of the catalytic domain both alone and in complex with small-molecule inhibitors (Andersen *et al.*, 2008 ▶; D’Alise *et al.*, 2008 ▶; Girdler *et al.*, 2008 ▶; Sessa *et al.*, 2005 ▶). Although this segment of INCENP does not perfectly match the Aurora B consensus motif (Alexander *et al.*, 2011 ▶), it is highly enriched with positively charged residues promoting the docking of the INCENP stretch onto Aurora B (Figs. 2 ▶ *a* and 2 ▶ *b*). During the structure refinement, we were intrigued by the precise docking of the INCENP C-terminus onto the Aurora B substrate-recognition region. Multiple sequence alignment revealed high conservation of the residues preceding the well conserved Thr848-Ser849-Ser850^INCENP^ (TSS) motif (Fig. 2 ▶ *c*), which is absent in our crystallization construct and has been shown to be crucial to achieve full Aurora B activity (Honda *et al.*, 2003 ▶; Sessa *et al.*, 2005 ▶). Of particular interest is the well ordered Arg847^INCENP^ residue deeply anchored into a pocket formed between pThr248^Aurora B^ and the αC helix, which is able to displace the side chain of the Arg140^Aurora B^ residue that in turn is involved in pThr248^Aurora B^ phosphate-group coordination (Fig. 2 ▶ *b*). A careful inspection of the Aurora B–INCENP–AMP-PNP electron-density map revealed the presence of an arginine residue (Arg163^Aurora B^) which projects out from a neighbouring Aurora B molecule, coordinated by a negatively charged pocket (mainly Glu281^Aurora B^; Figs. 2 ▶ *b* and 2 ▶ *d*). Further interactions arise from the nucleotide phosphate β group coordinating the positively charged residue Lys839^INCENP^ and Lys841^INCENP^ into the ATP-binding pocket (Figs. 2 ▶ *b* and 2 ▶ *d*), thus contributing with Arg847^INCENP^ to stabilization of the INCENP peptide on the Aurora B surface.

### 3.4. Comparison with protein kinase A in complex with the inhibitor peptide PKI   

Although unexpected, the crystal contact favoured by the presence of a nucleotide within the Aurora B ATP-binding cleft may resemble the inhibitory mechanism observed for protein kinase A (PKA), in which an inhibitor protein (PKI) was originally co-discovered (Walsh *et al.*, 1971 ▶). Interestingly, it was discovered to act by competitively binding with the protein substrate to the catalytic subunit of the protein kinase, binding with high affinity in an ATP-dependent manner (Ashby & Walsh, 1973 ▶). The structure of protein kinase A (PKA) was first reported in complex with the inhibitor peptide PKI, in which the position P(0) is occupied by an alanine (Knighton *et al.*, 1991 ▶). The residues in positions P(−3), P(−2) and P(−1) of PKI bind to the C-lobe of the kinase in a similar orientation to that observed for the INCENP segment bound to the Aurora B C-lobe (Figs. 2 ▶ *d* and 2 ▶ *e*). In support of this hypothesis, it has been reported that high local concentrations of Aurora B are required to obtain transphos­phorylation on the T-loop and thus full Aurora B activity (Kelly *et al.*, 2007 ▶). A mechanism for modulating the catalytic activity of Aurora B through a mimicking substrate-inhibitory strategy in cellular compartments where the enzyme is highly enriched is therefore not unreasonable (Fig. 2 ▶ *f*). Furthermore, the adjacent phosphorylatable TSS segment may play a role in relieving this transinhibitory mechanism (Fig. 2 ▶ *f*). Phosphorylation of the TSS motif is indeed required for full Aurora B activity (Bolton *et al.*, 2002 ▶; Bishop & Schumacher, 2002 ▶; Honda *et al.*, 2003 ▶; Sessa *et al.*, 2005 ▶). Our structural data predict that phosphorylation of the TSS motif would disfavour its proximity to the pThr248^Aurora B^ residue on the activation loop. In this model, the phosphorylated TSS motif of INCENP could reverse the inhibitory substrate-mimicking effects, generating a fully active kinase (Fig. 2 ▶ *f*). This model would explain the still unknown role of the phospho-TSS motif in achieving full Aurora B activity. Counteracting Ser/Thr phosphatases could then limit Aurora B activity when enriched at centromeres and/or kinetocores (*i.e* PP1 and PP2A phosphatases; DeLuca *et al.*, 2011 ▶; Foley *et al.*, 2011 ▶; Fig. 2 ▶ *f*). These data also provide useful insights for the design of Aurora-specific competitive synthetic peptide inhibitors. These pseudosubstrate inhibitor peptides could represent a valid and specific alternative to ATP-competitor small molecules.

## Supplementary Material

PDB reference: Aurora B–INCENP–barasertib, 4c2v

PDB reference: Aurora B–INCENP–AMP-PNP, 4c2w

## Figures and Tables

**Table 1:** Data-collection and refinement statistics Values in parentheses are for the outer resolution shell.

	Aurora BINCENPbarasertib	Aurora BINCENPAMP-PNP
Data collection		
Space group	*P*2_1_	*P*2_1_
Beamline	PXIII, SLS	PXIII, SLS
Unit-cell parameters		
*a* ()	46.06	45.92
*b* ()	68.10	66.65
*c* ()	117.08	116.90
()	96.65	96.83
Resolution ()	381.49 (1.531.49)	431.7 (1.741.70)
Unique reflections	114953	74748
Data completeness (%)	98.5 (80.3)	97.02 (93.53)
*R*_merge_ † (%)	4.9 (46.9)	3.1 (13.9)
*I*/(*I*)	14 (2.6)	30 (10)
Refinement		
Resolution range ()	381.49	431.7
*R*_conv_ ‡/*R*_free_ § (%)	16.6/19.7	15.6/19.5
No. of atoms in refinement	6022	6091
R.m.s.d., bond lengths ()	0.025	0.020
R.m.s.d., bond angles ()	2.370	2.198
Mean *B* factor (^2^)	23.43	23.23
Ramachandran statistics (%)		
Preferred regions	96.6	97.6
Allowed regions	3.2	2.4
Outliers	0.2	0
PDB code	4c2v	4c2w

† *R*_merge_ = 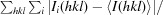 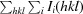, where *I_i_*(*hkl*) is the *i*th observed intensity of a reflection and *I*(*hkl*) is the average intensity obtained from multiple observations of symmetry-related reflections.

‡ *R*_conv_ = 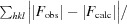 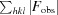, where *F*_obs_ and *F*_calc_ are the observed and calculated structure-factor amplitudes, respectively.

§ *R*_free_ is equivalent to *R*_conv_ for a 5% subset of reflections that were not used in the refinement.
